# Towards the Forest Virome: High-Throughput Sequencing Drastically Expands Our Understanding on Virosphere in Temperate Forest Ecosystems

**DOI:** 10.3390/microorganisms9081730

**Published:** 2021-08-14

**Authors:** Artemis Rumbou, Eeva J. Vainio, Carmen Büttner

**Affiliations:** 1 Faculty of Life Sciences, Albrecht Daniel Thaer-Institute of Agricultural and Horticultural Sciences, Humboldt-Universität zu Berlin, 14195 Berlin, Germany; carmen.buettner@agrar.hu-berlin.de; 2 Natural Resources Institute Finland, Forest Health and Biodiversity, Latokartanonkaari 9, 00790 Helsinki, Finland; eeva.vainio@luke.fi

**Keywords:** forest virome, high-throughput sequencing, mycoviruses, plant pathogenic viruses

## Abstract

Thanks to the development of HTS technologies, a vast amount of genetic information on the virosphere of temperate forests has been gained in the last seven years. To estimate the qualitative/quantitative impact of HTS on forest virology, we have summarized viruses affecting major tree/shrub species and their fungal associates, including fungal plant pathogens, mutualists and saprotrophs. The contribution of HTS methods is extremely significant for forest virology. Reviewed data on viral presence in holobionts allowed us a first attempt to address the role of virome in holobionts. Forest health is dependent on the variability of microorganisms interacting with the host tree/holobiont; symbiotic microbiota and pathogens engage in a permanent interplay, which influences the host. Through virus–virus interplays synergistic or antagonistic relations may evolve, which may drastically affect the health of the holobiont. Novel insights of these interplays may allow practical applications for forest plant protection based on endophytes and mycovirus biocontrol agents. The current analysis is conceived in light of the prospect that novel viruses may initiate an emergent infectious disease and that measures for the avoidance of future outbreaks in forests should be considered.

## 1. Introduction

Forests represent a major natural resource and provider of ecosystem services, products and jobs in Europe. They cover 35% of Europe’s total land area [1], their ecological importance being as significant as their economic importance. The majority of the countries have 30–45% of their land area covered with forests, while countries in northern Europe have larger forest areas, with three-quarters of the total land area in Finland and 69% in Sweden being covered by forests. The forest sector, consisting of the wood and paper industries contributes 1.97% of the total gross domestic product (GDP) in northern Europe and on average 0.72% of the total GDP of Europe [1,2]. Pests and diseases have been reported as important causes of damage among wildlife and grazing by domestic animals, fires and weather extremes, such as storms. Insect attacks, weather extremes and fungal diseases have been reported as the most common and widespread factors associated with tree defoliation [1]. Forest health is a “public good”, and multiple categories of end-users may benefit from it. The forest industry, seed producers, nurseries, producers of non-timber products and the broader society are end-users of forests and urban green areas as providers of multiple ecosystem services. The need of indicators to provide information on forest ecosystem health and vitality, which may enable an evaluation of its resilience, is clearly underlined [2].

Land plants are known to own a virome of a distinct composition, heavily dominated by diverse positive sense RNA ((+)RNA) viruses, with a more limited representation of double-stranded RNA (dsRNA), negative sense RNA ((−)RNA) viruses, reverse-transcribing (RT) viruses and single-stranded DNA (ssDNA) viruses, to the exclusion of bona fide dsDNA viruses [3]. The virome of land plants fits into two of the four realms, Riboviria—into which the majority of plant virome diversity fits—and Monodnaviria. Within Riboviria, two kingdoms are included. The first kingdom is Orthornavirae and harbors the bona fide RNA viruses with no DNA stage in their replication cycles and replication modules organized around the RNA-dependent RNA polymerase (RdRP). The second kingdom, Pararnavira, consists of reverse-transcribing viruses encoding a reverse transcriptase (RT), which is homologous to the RdRPs of RNA viruses. Viruses with ssDNA genomes encoding rolling-circle replication endonucleases (RCRE) of the HUH superfamily are classified into the realm Monodnaviria. The virome of fungi has a similar composition, lacking dsDNA viruses as well but exhibiting a bias toward dsRNA viruses [4]. According to the status of knowledge when the review of Ghabrial et al. [4] was published, fungal viruses made of linear dsRNA were classified into seven families: *Chryso-, Endorna- Megabirna-, Quadri-, Partiti-, Reo-* and *Totiviridae*. The linear (+)ssRNA viruses were classified into five families: *Alphaflexi-, Barna-, Gammaflexi-, Hypo-* and *Narnaviridae*. The linear (–)ssRNA belonged to the proposed family “Mycomononegaviridae”, while the circular ssDNA viruses were still unclassified.

Virus diseases of forest tree species represent a threat for the forest health that has been underestimated thus far [5,6]. Unfortunately, numerical data on the losses from virus diseased forest trees are lacking, but they are definitely assumed to be considerable based on experience from virus infection on fruit trees, while current data on symptom appearance, disease dispersal and epidemic severity provide essential evidence of this. Forest tree viruses are ranking highly in regard to their risk for introduction and establishment in forests and crops, and this lies mainly upon the wide distribution of susceptible plant germplasm, the often extended host range and the rapid emergence of new genotypes (virus strains or variants). Most common reports in the Program for Monitoring Emerging Diseases (ProMED) run by the International Society for Infectious Diseases (ISID) are reports of newly arrived viruses and viroids (36.6%) among approximately 140 emerging infectious diseases (EIDs) in plants [7]. From our own investigations and existing references, we suggest that viruses remarkably contribute to emerging infectious diseases and confirm Büttner and Nienhaus [5] who raised awareness of the issue in the 1980s.

Due to the utilization of high-throughput sequencing (HTS) methodologies, forest virology has gained a significant momentum in identifying viruses-infecting forest trees and associated fungi [8,9,10,11]. These platforms offer the possibility of metagenomic analysis, the study of microbial populations in samples by analyzing their nucleotide sequence content. RNA viruses, viroids and the RNA stages of actively replicating DNA viruses can be directly sequenced. Among HTS platforms employed, RNA-Seq and double-stranded RNA (dsRNA) sequencing of affected plant or fungal cells were most commonly applied; there were also, however, some cases where air metagenomis was performed or data were retrieved from a transcriptome shotgun assembly. Concretely, the investigation of a serious epidemic related to the birch leaf-roll disease (BLRD) in *Betula* spp. in Europe [12,13] represents a recent example of HTS application in forest virology. Employing RNA-Seq, birch viromes in relation with BLRD were unraveled revealing a complex of novel and known viruses (*Badna-, Nepo-* and *Capillovirus*) [14], while a novel badna virus was associated with the BLRD symptoms and was genetically characterized [15]. Another current viral epidemic in Europe is correlated with the European mountain ash ringspot-associated virus (EMARaV) of the genus *Emaravirus* within the family *Fimoviridae,* which is the causal agent of the “European mountain ash ringspot disease” in *Sorbus aucuparia* [16,17]. Regarding EMARaV, it was initially reported only in European mountain ash; lately, however, and with the use of HTS, several emaraviruses have been detected in new hosts, such as *Karpatiosorbus x hybrid* in Finland [18], *Sorbus intermedia* in Sweden [19] and *Amelanchier* spp. in Germany [20].

Regarding mycoviruses, a significant amount of data derive from the last decade through metagenomic analyses [21]. These viruses were not targeted earlier as, based on the traditional principles of phytopathology, they were not considered as non-plant pathogens. Viruses of the family *Partitiviridae* have also been largely ignored as they maintain a persistent lifestyle and they do not generally cause plant diseases. These are characterized by vertical transmission through seeds and/or pollen and a lack of pathogenicity and infectivity (ability to infect new hosts de novo via plant-to-plant transmission) [22]. The tree host in this review is considered as a holobiont, defined as the tree/shrub organism and all its microbiome, explicitly, symbiotic microbiota and pathogenic fraction (bacteria, oomycetes, protists and viruses) (definition by Margulis, 1993; [23]). Viruses that reduce the pathogenicity or growth of fungal plant pathogens can be considered beneficial for the holobiont, as they protect the tree from disease. In turn, viruses that would cause the debilitation of mutualistic mycorrhizal fungi are harmful from the viewpoint of the host plant. Therefore, mycoviruses will be addressed here based on the ecological guild of the host fungus: viruses of plant pathogens and viruses of mutualistic fungi.

This review aims to describe and comprehend the current status of the virosphere of a forest by summarizing the viral species coexisting in a forest. We name this “forest virome” and consider all existing genetic information of viral origin to be related to common forest trees and shrubs. Although the interactions between the host and viral agent are not always adequately defined, we have considered the following categories of interactions: a. pathogenic virus versus tree/shrub host, b. non-pathogenic virus versus tree/shrub host, c. pathogenic virus versus fungal host or d. non-pathogenic virus versus fungal host. In the term “forest”, we consider the natural temperate and boreal forest ecosystems in the Northern Hemisphere as well as urban parks and urban green areas mainly covered by forest trees and shrubs, which constitute separate ecosystems within urban environments. However, it is beyond the aim of the present review to list all studies performed to date; this work has been thoroughly carried out previously [6]. The aim of the present work is to update the list of pathogens considered as present in the forest ecosystem in light of the sequencing efforts and discoveries achieved in the last seven years. Regarding our aim to describe the forest virome, here, we do not consider observations that lack genetic characterization and sequence data availability.

The need for a current review about forest viruses is further strengthened by the fact that advances in plant virology are traditionally restricted in crop or fruit tree pathogenic viruses. This becomes apparent when considering the latest reviews [24,25] resuming viral discoveries using deep sequencing techniques in fruit trees, where virus discoveries in the forest ecosystem are not included. It is true that few groups worldwide focus their plant pathology research on forest ecosystems, which may be partially attributed to the difficulties in quantitatively estimating the importance of forest ecosystems and the economic impact due to viral epidemics. Despite this fact, it is shown through the present work that substantial progress has been achieved in this field, and in many cases, this has radically changed our understanding of viral forest diseases.

Finally, this review presents various lines of evidence regarding why further research on forest virome is required in order to a. biologically characterize the newly described pathogens, including their modes of dispersal and possible vectors; b. build the knowledge base of virus–tree interactions as well as of virus–fungus interactions; c. gather knowledge on the behavior of the forest parasites, in preparation for future outbreaks as a consequence of significant changes in the climate and the environment; d. explore alternative means of controlling fungal and viral forest diseases. As our understanding of the viruses of fungal endophytes and forest pathogens is only at the very beginning, it is an ideal time to apply the most modern sequenced-based tools in favor of forest virology.

## 2. Interaction of Viral Agents with Other Organisms

The current analysis was based on two main principles. The first was the hologenome theory of evolution [26,27]. According to this theory, a holobiont is a single dynamic entity in which a vast amount of its genetic information and variability is contributed by the microorganisms [28]. In this study, we considered a forest tree/shrub as a holobiont and focused on a part of its microbiome, the virome, considering all virus communities (as dependent biological agents) a. affecting the tree (plant pathogenic or latent viruses) and b. affecting the exosymbionts and endosymbionts of the tree (mycoviruses). The second principle was that “forest” was considered as an ecosystem with multiple holobionts (diverse tree species); our focus was put on the viral variation that characterizes the whole system, and this we called the “forest virome”.

To explore the studies involved in the described subject, we first listed viruses alphabetically, ordering them in three categories: 1. plant viruses infecting tree/shrub hosts and cryptoviruses (Table 1), 2. mycoviruses occurring in plant pathogenic fungi (Table 2) and 3. mycoviruses occurring in mutualistic fungi and saprotrophs (Table 3). To systematically handle the data, we listed in the following section the 11 most important forest tree or shrub genera known to be affected by viruses; a separate section is devoted to the virome of each plant genus (Section 3.1, Section 3.2, Section 3.3, Section 3.4, Section 3.5, Section 3.6, Section 3.7, Section 3.8, Section 3.9, Section 3.10, Section 3.11). Another section is devoted to “other trees”, where less information is available thus far (Section 3.12). For all searches to detect all related articles, the search-machine of Elsevier’s Scopus, a large citation database of peer-reviewed literature, was applied (www.scopus.com/search/; accessed on 20 June 2021).

### 2.1. Plant Viruses Infecting Tree/Shrub Hosts and Cryptoviruses

In the present review, we listed 43 plant viruses that affect 19 forest tree and shrub species present in the temperate forest and urban zone (Table 1 and references). The majority of these viruses are (+)ssRNA viruses of the families *Betaflexiviridae* (11 viruses), *Secoviridae* (seven viruses), *Bromoviridae* (five viruses), *Tombusviridae* (four viruses), *Virgaviridae* (three viruses) and *Mayoviridae* (one virus). Apart from the (+)ssRNA viruses, there is also a genus of (–)ssRNA, the *Emaravirus*, which is represented in the forest virome with four different species. There are also two reverse-transcribing dsDNA viruses: the birch leafroll-associated virus (BLRaV) in *Betula* spp. and the chestnut mosaic virus in *Castanea sativa*, both belonging to the *Badnavirus* genus (Family *Caulimoviridae*). Additionally, the first genetic evidence of the presence of another reverse-transcribing DNA virus has been reported in white ash (*Fraxinus americana*). Finally, three dsRNA viruses have been also detected in pines and in ash trees, two from the family *Partitiviridae* and one from *Amalgaviridae*.

In total, 21 viruses have been newly discovered within the last five years by applying HTS methodologies, which represent 49% of the total number of viral species in forest trees. Among these, three virus genera are overwhelmingly represented. These are the genus *Emaravirus* with four species to date (aspen mosaic-associated virus [42], common oak ringspot-associated virus [56,57], maple mottle-associated virus [74] and European mountain ash ringspot-associated virus [16]), the genus *Badnavirus* with two species (birch leafroll-associated virus [15] and chestnut mosaic virus [55]) and the genus *Carlavirus* with eight species (birch carlavirus [14], blueberry scorch virus [43,44], elderberry carlaviruses A, B, C, D, E [59,60] and elm carlavirus [61,62,63]). All the novel emara- and badnaviruses are found to be associated with the corresponding symptoms and are, consequently, plant pathogenic, while the role of carlaviruses is not yet clarified. Apart from these, single species are discovered from four other genera with (+)ssRNA: an *Idaeovirus* (unassigned species and family), a *Capillovirus* (*Betaflexiviridae*), a *Bromovirus* (*Bromoviridae*) and a *Aureusvirus* (*Tombusviridae*).

From the current knowledge, the majority of viruses detected in forest/shrub tree species exhibit a broad host range and were isolated in many other crops or trees (apple, cherry, tomato, tobacco, strawberry, blueberry, etc.) before their discovery in forest trees, but our aim is to reform this knowledge base, as there is a remarkably increasing number of novel viruses. The 21 novel viruses discovered through HTS to date are host specific and, following the nomenclature principles, the host name is included in the new species name. As a result, all novel virus species are named after their forest tree host. Only four viruses were named after a forest tree name before the discovery of the HTS: the poplar mosaic virus, the European mountain ash rinspot-associated virus, the elm mottle virus and the white ash mosaic virus.

### 2.2. Mycoviruses Occurring in Plant Pathogenic Fungi

In this study, we have summarized 63 viral species infecting ten highly important fungal or oomycete pathogens occurring in more than 11 genera of forest trees (Table 2 and references). In contrast to the plant pathogenic viruses, this group of viruses includes many dsRNA viruses mainly affiliated with the *Partitiviridae* family (17 viruses) but also families *Totiviridae* (three viruses) and *Curvulaviridae* (two viruses), while the family *Reoviridae* was represented by one virus species. As dsRNA viruses can be readily detected based on classical cellulose chromatography analysis, most of these viruses were already known before the employment of HTS methods. Similarly, some (+)ssRNA viruses, including members of *Hypoviridae*, *Endornaviridae* and *Mitoviridae,* had been already discovered prior to the HTS era based on their dsRNA replicative intermediates. HTS analysis has, however, revealed mitoviruses to be highly common in fungi, also in fungal species where members of this family were not earlier detected by classical virus screening methods. From the total of 33 (+)ssRNA viruses detected, 23 belong to the family *Mitoviridae;* 11 novel mitoviruses have been detected during the last five years applying HTS, while Cryphonectria parasitica mitovirus 1 [126], Fusarium circinatum mitoviruses [139], Gremmeniella mitoviruses 1 and 2 [142,143], Hymenoscyphus fraxineus mitovirus 1 [138] and Ophiostoma mitoviruses [150,151] were discovered earlier. Other (+)ssRNA viruses revealed by HTS methodology include a virga-like virus (unclassified *Virgaviridae*) and an ourmia-like virus (family *Botourmiaviridae*) in *Armillaria* spp. [116]. HTS methodology has also allowed for the first time the detection of (–)ssRNA RNA viruses in our target pathogens: Armillaria mellea negative strand RNA virus 1 [116] and Cryphonectria parasitica sclerotimonavirus 1 [125] (both putative members of *Mymonaviridae*). Finally, new unclassified ambisense viruses were very recently identified and characterized: Cryphonectria parasitica ambivirus 1- NB631 [125] and Armillaria ambi-like viruses [116]. Apart from the plant pathogenic fungi listed in Table 2, HTS has been shown to be a very efficient tool for the detection of viral diversity in the soilborne pathogen *Rosellinia necatrix* that infects many fruit trees and related natural trees, also leading to the discovery of members of *Hypoviridae*, as well as unclassified ”fusagraviruses“ and ”megatotiviruses“ [166]. Similarly, unclassified (+)ssRNA viruses of the proposed family ”Fusariviridae“ (proposal submitted to ICTV in June 2021) were discovered for the first time in *Gremmeniella abietina* by HTS [146].

The vast majority of fungal viruses are transmitted intracellularly, which enables them to spread without movement proteins and even without being protected by protein capsids. Due to this close host association and long co-evolution, most mycoviruses do not cause a major host debilitation. However, as described in more detail in the discussion below, there are several important exceptions where mycoviruses reduce the virulence or growth rate of their host and hence improve the health of the holobiont. The most important example is the chestnut blight pathogen, *Cryphonecria parasitica*, which can be controlled via the introduction of hypoviruses [167,168], while two alphapartitiviruses of *Heterobasidion* spp. [169] and a mycoreovirus of *Rosellinia necatrix* have also shown promising results [170,171] (see discussion). However, the outcome of a mycovirus–host association is complex and may be dependent on environmental conditions, interacting fungi or coinfecting viruses [130,147,169].

### 2.3. Mycoviruses Occurring in Mutualistic Fungi and Saprotrophs

In Table 3, we have summarized 13 viral species infecting eleven species of ectomycorrhizal (ECM) fungi associated with both broadleaved and conifer trees. Again, dsRNA viruses predominate; most of them belong to the family *Partitiviridae* (four viruses) and the rest to the families *Curvulaviridae* (two viruses) and *Totiviridae* (one virus). Thanks to HTS, novel dsRNA viruses were detected for first time in several species of ECM fungi in Turkey: *Picoa juniperi* (dsRNA virus of the proposed family Megatotiviridae, [160]), *Sarcosphaera coronaria* (putative *Partitiviridae* member, [165]), *Hygrophorus penarioides* (partitivirus, [155]), and in the false morel mushroom *Gyromitra esculenta* [158]. Regarding ssRNA(+) viruses, they had already been identified with traditional methods, including putative members of *Mitoviridae* (three viruses) and *Endornaviridae* (two viruses). For the first time, a mitovirus was, by means of HTS, fully genetically characterized in the ectomycorrhizal fungus *Geopora sumneriana* [156].

The first viruses infecting ECM fungi were discovered in commercially valuable truffles (genus *Tuber*), and only recently, viral diversity in mycorrhizal fungi has been addressed more from an ecological viewpoint. While HTS studies have already revealed hitherto unknown evolutionary trajectories and new viral genome organizations in ECM fungi and other mycorrhizae [172], the phenotypic effects of most of these viruses have not been systematically studied.

Saprotrophs that consume coarse or fine woody debris are somewhat less intimately connected with the plant host than mycorrhizal fungi and pathogens, and most of them cannot be connected to a single genus of host trees. Some of them produce edible fruiting bodies and for this reason have been subjected to virus screening studies by HTS. The shiitake mushroom (*Lentinula edodes*) is found on many broadleaved tree species, and although virus-like particles were found in this species already in the 1970s, HTS has recently revealed many new virus taxa, most notably, (–)ssRNA viruses not detected by traditional methods [173,174]. Very recently, a novel (–)ssRNA virus of the family *Mymonaviridae* and a partitivirus were also identified with the use of RNA-Seq in the *Bondarzewia berkeleyi* from oak wood [175]. Other important wood decay fungi known to be infected by symptomatic viruses but not yet subjected to HTS include, for example, the enokitake (*Flammulina velutipes*) [176] and the oyster mushroom (*Pleurotus ostreatus*) [177], favouring broadleaved trees.

## 3. Virome of Specific Plant Hosts

### 3.1. Acer *spp.*

Arabis mosaic virus, cucumber mosaic virus, maple mottle-mssociated virus

Maples are abundantly found in temperate forests and urban parks, with the most common species, *A. pseudoplatanus* (sycamore) and *A. platanoides* (Norway maple), representing a natural component of birch (*Betula* spp.) and fir (*Abies* spp.) forests [178]. With a natural distribution across the globe, maples occur in many habitats from the high altitudes of the Himalayas, to the rainforests of South East Asia, to rocky cliffs in the Mediterranean and the edge of swamps in N. America. In addition to the horticultural uses and the tourism related value of maples, they are also of importance to the timber industry and valuable as a food. A number of the larger maples are commercially grown for timber in N. America and Europe: *A. saccharum, A. nigrum, A. negundo, A. rubrum, A. pseudoplatanus*. Maple sugar and maple syrup can be primarily produced from sugar maple (*A. saccharum*), black maple (*A. nigrum*) and manitoba maple (*A. negundo*) [179]. As with many other tree species, maples are under threat in the wild primarily as a result of forest degradation and destruction. Global climate change adds further pressure to those maples that are naturally rare or restricted to high elevations. Out of a total of 191 maples assessed by the IUCN/SSC Global Tree Specialist Group, 83 are considered threatened with extinction at the global scale and therefore require conservation action [178].

Viral diseases in different maple species have since long been reported [180,181]. Arabis mosaic virus [40,41] has been reported to infect *Acer* spp. However, until recently, maple has never been unambiguously described as a host for any well-characterized viral agent. Employing HTS methodology, a novel emaravirus was recently reported in sycamore maple (*A. pseudoplatanus*) exhibiting leaf mottle symptoms in Germany and was genetically characterized [74]. RNA-Seq was performed on the Illumina HiSeq2500 system using RNA preparations from symptomatic and symptomless maple trees. The sequence assembly and analysis revealed the presence of six genomic RNA segments in the symptomatic sample; RNA1 encoding the viral replicase (RdRP), RNA2 encoding the glycoprotein precursor (GPP), RNA3 encoding the nucleocapsid protein (N), RNA4 encoding the putative movement protein (MP) and RNA5 and RNA6 encoding proteins of unknown function. The novel virus was named maple mottle-associated virus (MaMaV), and evidence is provided that it may be the symptom-inducing virus in the diseased trees. The maple virome of the symptomatic tree tested was found to be very simple, as it included a single variant from a single virus. The lack of virome complexity is rather surprising, when we consider the HTS results obtained from other wild as well as cultivated woody hosts. A possible explanation could be the age of the tree; it was 3 years old when it was sampled, thus it was exposed for only a short time to viral pathogens [74].

### 3.2. Betula *spp.*

apple mosaic virus; arabis mosaic virus; birch leafroll-associated virus; birch capillo-virus; birch carlavirus; birch idaeovirus; cherry leaf roll virus; Phytophthora cactorum RNA virus 1; tobacco necrosis virus; tomato ringspot virus

Birches are an essential ecological component in northern temperate and boreal forests [182]. They are light-demanding early successional pioneer species, which rapidly occupy open areas after forest fires and clear-cuttings due to their prolific seed production and fast juvenile growth [183]. For forestry, birch is the most important broadleaved tree species in northern and eastern Europe. In Nordic countries, the proportion of birch out of the total volume of the growing stock varies between 11 and 16% and in Baltic countries, 17 and 28% [184]. Its ecological impact is unique, as it constitutes part of the few remaining old-growth forests growing in Europe, the proportion of which is rapidly decreasing across the entire boreal zone. Additionally, it contributes to forest resilience and the rapid restoration of wood production after disturbance by colonizing forest gaps and quickly increasing soil functioning and biodiversity [183]. Regarding biodiversity, the number of specialized flora and fauna species associated with birch is higher than for other tree species in Europe, particularly for mycorrhiza and insects. Furthermore, birch is adaptable to diverse climate conditions and can be integrated in diverse productive mixed tree stands, being, therefore, an appropriate forest species under current circumstances of rapid climate change. Concretely, regarding *B. pedula* and *B. pubescens*, due to the numerous strengths and potentials of these tree species, it is recommended by Dubois et al. [184] to expand its use in the Western European forestry considering societal, ecological, and economic purposes in a changing climatic and socio-economic context.

Several common tree viruses have been since long reported in birch: apple mosaic virus [31,34], arabis mosaic virus [35], cherry leaf roll virus [46], tobacco necrosis virus [35] and tomato ringspot virus [35]. In Finland, a severe birch decline was first observed in 2002 involving leaf symptoms such as vein banding, leaf roll, chlorosis and subsequent necrosis, causing a loss of vigor and degeneration in the trees. The disease has been widely distributed the last two decades, so far being reported in five European countries with diverse climate conditions (Finland, Sweden, Norway, Germany and France) [13,185,186,187,188]. The emerging phenomenon was described as “birch leaf-roll disease” (BLRD) [12,187] and was initially related to the presence of cherry leaf roll virus (CLRV) in the affected trees—based on standard molecular diagnostic tools [12,13,188]. However, the employment of HTS radically changed our concept regarding the causal agent of the BLRD. RNA-Seq revealed the presence of a novel badnavirus in affected birches [15], while a complex virome involving novel and known viruses was revealed; apart from the novel badnavirus, birch leafroll-associated virus (BLRaV), also birch idaeovirus, birch capillovirus, birch carlavirus and cherry leaf roll virus were present in symptomatic trees [14]. Interestingly, in single hosts, the virome could be highly variable, with up to five viruses and different variants of BLRaV and CLRV being detected in one sample. Based on the metagenomic analysis of three birches that exhibited symptoms and two that did not exhibit symptoms and on further analyzing a considerable number of samples [189], it is suggested that BLRaV is the main causal agent of BLRD; however, the other viruses could possibly still contribute to symptom development in cases of mixed infection with BLRaV and/or CLRV.

The oomycete *Phytophthora cactorum* is a birch pathogen that causes stem lesions and infects mostly nursery seedlings [190]. Besides birch, it infects over 200 species of trees, ornamentals and fruit crops. A recent HTS study revealed alphaendornaviruses, bunya-like viruses, a toti-like virus and viruses affiliated to the unclassified dsRNA virus group tentatively called “ustiviruses” in *P. cactorum* isolated from strawberry plants [153]. Furthermore, birch may be infected with *Armillaria* root rot fungi, which typically attack trees weakened by drought or other pathogens. A recent NGS study detected a novel virus group called “ambiviruses” in *Armillaria borealis* growing on birch wood [116].

### 3.3. Castanea Sativa

chestnut mosaic virus, Cryphonectria hypovirus 1, mycoreovirus 1

The sweet chestnut (*Castanea sativa*) is the only native species of the genus in Europe [191]. The broad diffusion and active management by man resulted in the establishment of the species at the limits of its potential ecological range. The present distribution ranges from North-Western Africa (e.g., Morocco) to North-Western Europe (southern England, Belgium) and from south-western Asia (e.g., Turkey) to Eastern Europe (e.g., Romania), the Caucasus (Georgia, Armenia) and the Caspian Sea. The sweet chestnut has a remarkable multipurpose character and may be managed for timber production (coppice and high forest) as well as for fruit production (traditional orchards), including a broad range of secondary products and ecosystem services [191].

Chestnut mosaic disease (ChMD) represents one more case in the forest pathology, where knowledge concerning the causal agent was gained thanks to HTS and bioinformatics analysis. The disease was described several decades ago in Italy [192] associated with viral symptoms, such as mosaic and shoots with asymmetric leaf blade deformation. In 1987, it was reported in France [193], involving necrotic lesions in the bark and wood that turn into cankers, chlorotic lesions and yellow stripes on leaf veins and partial limb atrophy, thus heavily affecting the production [194]. By using RNA-Seq analysis, two independent isolates of the same novel virus were identified [55]. The novel chestnut mosaic virus belongs to the genus *Badnavirus*, family *Caulimoviridae*; it is unambiguously proven to be episomal and is strongly suggested to play causal role in the disease development.

Chestnut blight is another serious disease of chestnut caused by the ascomycete *Cryphonectria parasitica*. As stated above, hypoviruses infecting the pathogen have been successfully used to control the fungus, and the subsequent natural spread of these viruses has protected European chestnut trees from complete devastation [167,168]. Additionally, other viruses (including Mycoreovirus 1 and Cryphonectria mitovirus 1) infect this pathogen, and these have been recently examined for their biocontrol and transmission potential in planta. Some promise was shown, but the use of the viruses is highly dependent on their transmission efficacy [195].

### 3.4. Fraxinus *spp.*

arabis mosaic virus; cherry leaf roll virus; Hymenoscyphus fraxineus mitovirus 1; tobacco mocaic virus; tobacco necrosis virus; tobacco ringspot virus; white ash mosaic vi-rus; putative cryptovirus; putative caulimovirus

Although several viruses are reported in ash trees, all of them are generalists, and none of them is host specific. The most common are arabis mosaic virus [37,38], cherry leaf roll virus [47], tobacco necrosis virus [105], tobacco mosaic virus [97,98,99], tobacco ringspot virus [98,99,110], tomato ringspot virus [96] and white ash mosaic virus [114].

With the use of HTS, a novel putative partitivirus and a novel putative caulimovirus have been identified in *Fraxinus americana* [115], exhibiting symptoms distinct from those caused by previously reported ones, namely, chlorotic patches and necrotic lesions on leaves. Commonly, members of *Partitiviridae* are not pathogenic for their host; recently, however, new members of the family were found to be associated with symptom development. Nevertheless, further studies are needed in order to fully characterize the new viruses in ash.

A recently discovered mycovirus related to the ash microbiome/virome is Hymenoscyphus fraxineus mitovirus 1 (HfMV1) [138]. *H. fraxineus* is a pathogen recently introduced into Europe from Asia, causing ash dieback and threatening ash stands all across the continent [196]. *H. fraxineus* isolates from Europe were previously shown to harbor HfMV1, while later, a viral population with higher genetic diversity was detected in *H. albidus*, a harmless litter saprotroph native in Europe. This fact suggests multiple interspecific virus transfers from *H. albidus* to *H. fraxineus* [196].

### 3.5. Picea *spp.*

Heterobasidion mitovirus 1, 2 and 3; Heterobasidion RNA virus 6; Heterobasidion partitiviruses; Picea mariana tenuivirus; tomato mosaic virus; tomato mosaic virus

Concerning Norway spruce, it is a long-living species (>200 years-old) with a long tradition of cultivation for its straight trunk, particularly used for timber constructions, pulpwood for paper and furniture [197]. Its high economic and ecological significance calls for taking proactive measures against potential viral emergence. Starting from the 1980s, spruce forests have shown symptoms of decline in mountainous areas of central Europe, including yellowing, loss of needles, die-back of branches and reduced growth. Due to widespread spruce pathogens and pests, such as *Heterobasidion parviporum* and *Ips typographus*, as well as health problems of unidentified aetiology, its popularity for reforestation, particularly outside its natural range in central European forests, has been reduced, although it remains the most commercially valuable tree species in the Nordic countries.

From the plant pathogenic viruses, only tomato mosaic virus has been reported in spruce [93,94]. *Picea mariana* has been reported to host (–)ssRNA viruses of the genus *Tenuivirus* [198].

The most important fungal pathogen infecting Norway spruce is *Heterobasidion parviporum*, which causes economic losses of hundreds of millions of euros annually in Europe [199]. Like the related species, *H. annosum* prefers pines as its host, and *H. parviporum* hosts partiti-, curvula- and mitoviruses (Table 2). The Heterobasidion partitivirus 15 (HetPV15-pa1) of *H. parviporum* is associated with the debilitation of its fungal host [169]. The ectomycorrhizal symbionts of spruce include species of *Lactarius, L. tabidus* being one of the most common ones. This species also harbors members of the family *Curvulaviridae* (Table 3; [159]).

### 3.6. Pinus *spp.*

Gremmeniella abietina RNA virus MS1; Gremmeniella abietina RNA virus L1; Gremmeniella abietina mitochondrial RNA virus S1 and S2; Fusarium circinatum mi-tovirus 1, 2-1 and 2-2; Heterobasidion partitiviruses; Pinus nigra virus 1; Pinus patula amalgavirus 1; Pinus sylvestris partitivirus NL-2005; Sphaeropsis sapinea RNA virus 1; tobacco necrosis virus

*Pinus* spp. are attacked by various fungal pathogens, including the root rot fungi *Heterobasidion annosum* (Europe) and *H. irregulare* (North America and Europe as an introduced invasive species). These pine pathogens host viruses of the families *Partitiviridae* and *Mitoviridae,* and both virus families also have apparently cryptic members infecting plants ([137]; https://talk.ictvonline.org/, accessed on 26 July2021). The Heterobasidion partitivirus 13 (HetPV13-an1) of *H. annosum* is associated with host debilitation ([200]; see Discussion). *H. annosum* also hosts Heterobasidion RNA virus 6 (HetRV6), a member of a newly classified family, *Curvulaviridae* (Table 2; [129]). The spore-mediated dispersal of *H. annosum* and *H. parviporum* can be controlled by the preparation of the saprotroph *Phlebiopsis gigantea*, which acts as an antagonist to *Heterobasidion* spp. and occurs very commonly in newly cut conifer wood, especially Scots pine. *P. gigantea* can be infected by mycoviruses tentatively named “phlegiviruses” according to their host fungus [201].

Pines also suffer from needle diseases, such as *Scleroderris* cancer caused by *Gremmeniella abietina* and wilting disease caused by the globally spreading pathogen *Fusarium circinatum*. The ascomycete *G. abietina* causes shoot blight and stem canker of several conifers in Europe and N. America. The fungus hosts a diverse virus community (Table 2; [146]). One of the viruses, representing the virus family *Curvulaviridae*, has been associated with phenotypical changes in the host (enhanced mycelial growth) [144]. *F. circinatum* is also commonly infected with mitoviruses (Table 2, [139,140,141]). Viruses of the families *Totiviridae* and *Mitoviridae* also occur in the pine needle pathogens *Diplodia pinea* and *Cronartium ribicola*, respectively (Table 2).

On the other hand, pines have several fungi as their symbiotic partners form ectomycorrhizal associations. One of the most common is *Thelephora terrestris*, which hosts a “phlegivirus” that was also detected in soil oribatid mites, suggesting that even some RNA mycoviruses could have arthropod vectors [202]. Additionally, the basidiomycete *Lactarius rufus* is a highly common ectomycorrhizal symbiont of pine trees and has been shown to be commonly infected by members of the family *Curvulaviridae* (Table 3; [159]). Remarkably, a single ascocarp of the ectomycorrhizal crown cup fungus *Sarcosphaera coronaria* associated with *Pinus brutia* was shown to be infected with ~34 different partitiviruses [165]. The same research group found a partitivirus in another ectomycorrhizal partner of *P. brutia, Gyromitra esculenta* [158], which may also be infected by endornaviruses as revealed by the in silico analysis of transcriptomic datasets [157].

Not many plant pathogenic viruses from the *Pinus* trees themselves are known. One of the rare ones is the cryptovirus Pinus sylvestris partitivirus NL-2005 (Table 1, [81]). Additionally, Pinus patula amalgavirus 1 has been reported in a transcriptome shotgun assembly (TSA) database [80], illustrating the value of mining the TSA and other databases for novel viral sequences.

Pinus nigra virus 1, an unclassified *Caulimoviridae* member, was discovered through air metagenomics (using Illumina technology) in Spain and was later PCR-detected in *Pinus nigra* samples from the vicinity of where the air samples were collected. This suggests that this new virus is likely a pathogen of *Pinus* [79]. Another pathogenic virus reported in *Pinus* is tobacco necrosis virus [106], but it has not been further investigated.

### 3.7. Populus *spp.*

arabis mosaic virus; Armillaria borealis mycovirgavirus 1; Armillaria borealis am-bi-like virus 1 and 2; aspen mosaic-associated virus; poplar mosaic virus; tobacco necrosis vi-rus; tomato black ring virus

*Populus* is a tree native to most of the Northern Hemisphere including 25–30 species. Six of these aspen species play a disproportionately important role in promoting biodiversity, sequestering carbon, limiting forest disturbances and providing other ecosystem services [203]. Importantly, aspen species are commonly designated “keystone species”, meaning their sustained existence supports an inordinate number of dependent plants and animals.

Regarding viruses affecting poplars, four viruses have been previously identified: arabis mosaic virus [36], tomato black ring virus [36], tobacco necrosis virus [106] and poplar mosaic virus [82,83,84]. The latter is one of the few viruses discovered in the before-HTS-era that is a specialist. Very recently, based on RNA-Seq analysis, a novel emaravirus was discovered in Eurasian aspen, named aspen mosaic-associated virus [42]. The monocistronic, segmented ssRNA genome of the virus shows a genome organization typical for emaraviruses encoding the viral RdRP on RNA1, a GPP on RNA2, the viral N on RNA3 and a putative MP on RNA4. The fifth identified genome segment (P28) encodes a protein of unknown function. The virus is closely associated with observed leaf symptoms, such as mottle, yellow blotching, variegation and chloroses along veins. Observed symptoms and testing of mosaic-diseased Eurasian aspen by RT-PCR confirmed a wide geographic distribution of the virus in Fennoscandia [42].

Poplars, as many other hardwood trees, are also readily infected by species of *Armillaria*. One of the most common species is *A. borealis* occurring in both conifers and hardwoods. Recently, HTS analysis revealed a Siberian isolate of *A. borealis* from *Populus* spp. to be infected by multiple viruses, including a novel ssRNA(+) virus named Armillaria borealis mycovirgavirus 1 and three ambi-like viruses [116].

### 3.8. Quercus *spp.*

Armillaria mellea negative strand RNA virus 1; Armillaria mellea ourmia-like virus 2; common oak ringspot-associated virus; tobacco mocaic virus; tobacco necrosis virus

The genus *Quercus* contains over 500 species. Oaks are amongst the most economically and ecologically important deciduous trees in Europe providing wood for fuel, bark for tanning, timber for construction and acorns for livestock [204]. Here, we present existing virome data from *Q. robur* (common oak)*, Q. petraea, Q. variabilis* (chinese oak), and *Q. rubra* that are native in Europe, North America and Asia.

Apart from two viruses with wide host spectra reported to occur in oaks, tobacco necrosis virus [106] and tobacco mosaic virus [97,98,99,100,101,102,103,104], still the main virus disease affecting the species remained unidentified until recently. The “chlorotic ringspot” disease of oaks originating from the USA was reported in the 1970s in Europe [104], and its occurrence was estimated to be relatively high in Germany and Scandinavia [6]. The disease was also found to affect 11–19% of oak seedlings in propagation stations, threatening by wider spread of the disease through the infected oak propagation material [6]. Due to the employment of HTS strategies, the causal agent of the disease was recently identified; the common oak ringspot-associated virus (CORaV) [56,57] represents a new member of the genus *Emaravirus* comprising five RNA segments. Typically for emaraviruses, the RdRP is encoded by RNA 1, while RNA 2 encodes the GPP. The viral nucleocapsid protein (N) is encoded by RNA 3, and RNA 4 encodes the MP. RNA 5 of CORaV contains one major open reading frame, coding for a protein P5 of 179 aa; however, the function of P5 remains to be elucidated. This is one more occasion where metagenomic analysis provided the diagnostic answer to a problem that has remained unsolved for a long time.

Apart from the virus diseases, oaks also suffer from the “Sudden Oak Death” caused by *Phytophthora ramorum*—an oomycete that can kill oaks within a few weeks—and from “Oak Wilt”—caused by the fungus *Ceratocystis fagacearum*. The oak forest decline is a serious problem in North and South America. In the USA, entire oak ecosystems have declined due to a combination of factors that remain unclear [205]; it is, however, hypothesized that viral diseases are part of this complex syndrome. Notably, endornavirus strains have been found in *Phytophthora ramorum* isolates from various host plants, including *Rhododendron* and *Viburnum* species, both in the United States and Europe, but unfortunately, they do not seem to cause host debilitation that could be used for disease mitigation [152]. Already declining oak trees also readily suffer from infections by *Armillaria* root rot fungi. Recent discoveries of viruses in *Armillaria* spp. [116] also prompt the search of debilitation-associated viruses in these species. However, the viruses found thus far (Armillaria mellea negative strand RNA virus 1 and Armillaria mellea ourmia-like virus 2) in *A. mellea* from oak trees in South Africa did not seem to reduce the growth of their host in laboratory conditions.

Oaks are known to form mycorrhizal associations with both ascomycetes, such as true truffles (*Tuber* spp.) and *Cenococcum geophilus*, as well as basidiomycetes, such as species of *Lactarius, Russula* and *Cortinarius*. Viruses have been found in the ectomycorrhizal fungus *Hygrophorus penarioides*, but their possible effects on the tree-fungus association are unknown to date [155]. Moreover, *Tuber* spp. (*T. excavatum* and *T. aestivum*) have been shown to harbor diverse viruses of the families *Totiviridae, Endornaviridae* and *Mitoviridae* (Table 2).

### 3.9. Sambucus *spp.*

blueberry scorch virus; cherry leaf roll virus; cherry rasp leaf virus; elderberry au-reusvirus 1; elderberry carlavirus A, B, C, D and E; elderberry latent virus; Sambucus virus S; tobacco ringspot virus; tomato bushy stunt virus; tomato black ring virus

Elderberry (*Sambucus nigra* L.) is a deciduous tree native to Europe and North America. Its flowers and berries are used to prepare infusions, syrups and jellies and in traditional medicine. The popularity of this plant has increased in recent years in the pharmaceutical and food industries, due to its antiseptic and antiviral properties as well as to the interest in the colour compounds present in the berries [206].

From the abundant viruses affecting elderberry, eight are generalists and seven are specialists. Blueberry scorch virus [44], cherry leaf roll virus [49], cherry rasp leaf virus [54], tomato bushy stunt virus [50,91], tomato black ring virus [90] and tobacco ringspot virus [112] have been reported for a long time in elderberries and in other forest species worldwide. One of the specialists in this host is elderberry latent virus [49,59] reported in the USA. HTS technology contributed to our knowledge on viral diseases in elderberry with the discovery of seven host specific viruses. The elderberry aureusvirus 1 is either asymptomatic or associated with mild chlorotic mosaics and was detected by applying Illumina sequencing in dsRNAs [58]. Through dsRNAs Illumina sequencing the bromovirus sambucus virus S was also identified [85]. Five elderberry carlaviruses (elderberry virus A–E) were detected with the use of a degenerate oligonucleotide primed (DOP) RT-PCR method with multiple barcodes for HTS, involving VirFind, a novel and automated bioinformatics tool specifically used for virus detection and discovery [59,60].

### 3.10. Sorbus *spp.*

apple chlorotic leaf spot virus; apple mosaic virus; cherry leaf roll virus; European mountain ash ringspot-associated virus

The mountain-ashes are native throughout the cool temperate regions of the Northern Hemisphere, with the highest species diversity in the Himalaya. *S. aucuparia*, commonly called rowan, is an important deciduous tree or shrub, native to most of Europe and parts of Asia, as well as northern Africa. It serves as an ornamental urban species; it is also cultivated for its fruits and its timber [207].

The main disease affecting *S. aucuparia* is the “European mountain ash ringspot disease” caused by European mountain ash ringspot-associated virus (EMARaV), inducing chlorotic ringspots, mottle and line pattern on leaves and is widespread in central/northern Europe and England [16]. In addition to symptom development, EMARaV also causes symptomless infections; it is, therefore, assumed to be more common in the wild mountain ash trees than previously thought. Monitoring EMARaV in European forests revealed a very important aspect in viral epidemiology: the ability of viral pathogens to have an extended host range. Concretely, EMARaV was recently detected in new hosts, namely, in *Amelanchier* spp. in Germany [20], *Karpatiosorbus x hybrid* in Finland [18] and *Sorbus intermedia* in Sweden [19]. Concretely, in *S. intermedia* (Swedish whitebeam), it was revealed through high-throughput Illumina RNASeq that EMARaV in this host possesses two additional RNA segments, in contrast to the four RNAs known to be possessed by EMARaV in mountain ash [19]. The fifth genome segment identified in diseased whitebeam codes for a protein showing distant aa sequence identity to the functionally characterized MPs encoded by other putative MPs of similar size encoded by the RNA4 of other emaraviruses. This suggests that it is the functional orthologue responsible for cell-to-cell transport of the virus. The two additional genome segments were consistently detected in affected Swedish whitebeam as well as in diseased *S. aucuparia*, together with the previously known RNA1–RNA4 segments. The systematic presence of RNAs 5 and 6 in diseased *Sorbus* spp. and their absence in healthy trees suggest that the two newly identified genome segments encode proteins that are indispensable for the virus. Future studies are required to functionally confirm that the RNA5-encoded protein is the EMARaV MP and to investigate the role of the P27 encoded by the novel RNA6 in the life cycle of EMARaV.

Apart from EMARaV, only a few viruses have been detected in mountain ash: the apple mosaic virus [31], the apple chlorotic leaf spot virus [29] and the cherry leaf roll virus [51].

### 3.11. Ulmus *spp.*

cherry leaf roll virus; elm carlavirus; elm mottle virus; tomato ringspot virus; Ophi-ostoma mitoviruses

Several viruses have been reported to affect elms: elm mottle virus [64,65], tomato ringspot virus [95] and cherry leaf roll virus [51,208]. Recently, a disease affecting elms attributed to a non-characterized virus was investigated with the use of RNA-Seq methodology. This method revealed the presence of a novel carlavirus, the elm carlavirus [61,62,63], which is strongly suggested to be the causal agent of dieback and leaf symptoms, such as chlorotic ringspots, mottling and necroses in elms.

Mitoviruses are common in *Ophiostoma ulmi* and *O. novo-ulmi*, the causal agents of the Dutch elm disease (DED), devastating American elms but also causing epidemics in Europe and North America after new introductions. Some of these mitoviruses are associated with the reduced growth and sporulation of the host fungus [209]. Eight independently replicating mitoviruses were detected by Doherty et al. [150] in one diseased isolate of *O. novo-ulmi*, while two more mitoviruses were detected in a Canadian isolate of *O. novo-ulmi* [151]. The use of virus-induced hypovirulence as a biological control relies on the ability to transfer the virus between isolates within a population of the target pathogen. RNA viruses that have been found in *O. novo-ulmi* to date are located in mitochondria and can only be transmitted during anastomosis between compatible hyphae or induced forms of cytoplasmic mixing [151]. These findings raise the potential for engineering these viruses to include other genetic elements, such as anti-sense or interfering RNAs, to create novel and highly specific biological controls.

### 3.12. Other Tree Species

1. *Prunus* spp.: *Prunus* trees may suffer from infections by *Chondrostereum purpureum*, which causes silver leaf disease and has been developed as a biocontrol tool for the prevention of sprouting. The fungus hosts an alphapartitivirus called Chondrostereum cryptic virus 1 [210]. This tree genus may also be attacked by the notorious white root rot fungus *Rosellinia necatrix* that has a very broad host range, including both tropical and temperate fruit and forest trees.
2. *Aesculus* spp.: Some plant pathogenic viruses have been reported in *Aesculus* (apple chlorotic leaf spot virus, apple mosaic virus, cherry leaf roll virus and strawberry latent ringspot virus) (Table 1). Cryphonectria hypovirus 1, most commonly affecting *Cryphonectria parasitica* in chestnut, is also present in *Aesculus hippocastanum* (Table 2).
3. *Fagus* spp.: Earlier reports exist on the occurrence of cherry leaf roll virus and tobacco necrosis virus in beech. A recent RNA-Seq investigation revealed a novel carlavirus related to leaf symptoms in trees in Germany [62].
4. *Robinia* spp.: Strawberry latent ringspot virus and peanut stunt virus (Iran) (Table 1) have been reported.
5. *Salix* spp.: A few generalist viruses may occur in *Salix*, such as brome mosaic virus, tomato mosaic virus and tobacco necrosis virus.
6. *Cedrus libani*: The ectomycorrhizal ascomycete *Geopora sumneriana* is associated with Lebanon cedar. Recently, Geopora sumneriana mitovirus 1 was identified in this fungal species [156].
7. *Abies* spp.: No plant pathogen has been reported. Several partitivirus have been reported in *Heterobasidion* basidiomycetes infecting diverse *Abies* species: Heterobasidion partitivirus 1 in *A. cephalonica*; Heterobasidion partitivirus 10 in *A. concolor*; and Heterobasidion RNA virus 6 (an orthocurvulavirus) in *A. alba, A. sibirica, A. cephalonica, A. cilicica, A. equi-trojani* and *A. concolor* (Table 2).
8. *Pseudotsuga menziesii*: Aphaendornaviruses infect members of *Phytophthora ramorum* and *Phytophthora* taxon douglasfir [152].

## 4. Discussion

Based on the data presented in the current review, it has become obvious that HTS has considerably impacted discovery of novel viruses in organisms (trees and fungi) that contribute to what a forest is. Analogically, our knowledge regarding virus communities in urban parks has considerably increased. Almost half of the plant pathogenic viruses and cryptoviruses in plants (21 out of 43), 35% of the mycoviruses infecting pathogenic fungi (22 out of 63) and 54% of the mycoviruses occurring in non-pathogenic fungi (7 out of 13) were discovered due to the employment of HTS strategies. Specifically considering phytopathogenic viruses, novel viruses were not identified in random environmental or host samplings, but after targeting diseased trees, where the disease has been previously identified and the disease distribution was monitored for a long time period; however, the causal agent had not been identified due to limitations in the existing conventional molecular diagnostic methods. Concerning mycoviruses, even in species that have previously been extensively investigated, the HTS of a small number of isolates has revealed novel (+)ssRNA and (–)ssRNA viruses, which were viruses undetectable by traditional methods. The ability of HTS methods to extend the genetic investigation to a depth that had not been earlier achieved has resulted in extraordinary discoveries that have radically changed the view of forest pathology.

The vast majority of the viral families described in land plants are also shown to be present in the forest virome. Only viruses belonging to the realm Monodnaviria are not found in the forest, neither in tree nor in fungal hosts. Similarly to the taxonomical content of viruses described in plants, from the kingdom Orthornavirae, the (+)ssRNA viruses dominate the forest virome (members from seven families detected). Additionally, from the Orthornavirae, one virus genus of (–)ssRNA viruses, which are scarce in plants, the *Emaravirus* (*Fimoviridae*), and two families of dsRNA viruses (*Partitiviridae* and *Amalgaviridae*) were found, and this was achieved via HTS technologies. From the Pararnavira kingdom, only the family *Caulimoviridae* is represented with several RT viruses in the plant section of the forest virome. These are novel pathogenic viruses in birch, chestnut, pine and ash, which were unsuccessfully investigated with conventional diagnostic methods for many years, until HTS finally provided the solution. Concerning mycoviruses, analogously to land plants, dsRNA viruses also predominate in the forest, while RT viruses and circular ssDNA viruses have not yet been detected. By employing HTS, numerous novel dsRNA viruses, members of the families *Partitiviridae, Totiviridae,* Megatotiviridae (newly proposed family; [160]) and *Megabirnaviridae* (10^th^ report of ICTV; [211]) were characterized. The list of (+)ssRNA viruses was further extended with the recent HTS-generated data. It needs to be underlined that, according to ICTV, *Endornaviridae* is no longer a dsRNA, as previously described [4], but rather a family of (+)ssRNA viruses [212]. Apart from *Endorna*-, the families *Virga-, Botourmia, Mito- and Fusariviridae* are also newly represented in the forest mycovirome thanks to the discoveries of HTS. Furthermore, for the first time, (–)ssRNA viruses were detected in the fungal section of the forest virome: two of them belong to the family *Mymonaviridae* [213] and one belongs to the unclassified virus genus *Ambivirus* [125].

The massive discoveries of viruses in the forest ecosystem based on HTS technologies usually only refer to the full or partial genome sequence of the novel viruses, while knowledge on its biology and symptom expression is limited. To provide a basis for assessing the risk the novel viruses pose and make scientifically based decisions, a series of unavoidable steps need to be taken [214]. The early steps include the confirmation of infection using complementary methods; provisional taxonomy assignment; sample documentation adding information such as symptomatology and geographical origin; full genome sequencing and annotation (in cases of incomplete viral sequences); and the development of a diagnostic protocol accessible to all affected parties, as well as small-scale epidemiological surveys at the discovery location. The in-depth characterization is a mid- or long-term goal that involves the investigation of the virus pathogenicity, transmission experiments to explore modes of dispersal and possible vectors as well as large-scale surveys, organized on a national or international scale. A very interesting and universal technique for the in-depth characterization of a virus is the preparation of infectious clones. This requires the complete viral genome sequence, and it may offer valuable information about the ability of a virus to cause disease and the symptom development involved with the virus presence for individual viruses or for a mixture of viruses, while it can also support host range and transmissibility studies. This framework has already been actively followed by numerous research groups that focus on plant virology [215]. Regarding forest virology, however, as mentioned in the introduction, the progress is anticipated to be too slow, due to the few groups that focus on these issues, and if research in this field in not drastically financed, we may face unpleasant consequences in the future.

Based on the data of the last seven years, it has been revealed that the genetic variation of the forest virome is much larger than estimated. After gathering information about the viral presence in the holobionts, we could—as a next step—estimate the role of this virome in holobionts, the need for which was underlined by Rosenbeng and Zilber-Rosenberg [216]. One of the four basic principles of hologenome theory is that genetic variation in the hologenome can also be caused by changes in the microbiome genome [216]. In the following, we attempt to make suggestions on how the novel genetic information may qualitatively influence our view regarding forest health.

An important source of genetic variation is the horizontal gene transfer (HGT). As an example, 128 genes identified in the genome of moss *Physcomitrella patens* were acquired by HGT from prokaryotes, fungi or viruses [217]. Interestingly, many open reading frames (ORFs) showed high phylogenetic affinities to giant DNA viruses (nucleocytoplasmic large DNA viruses; NCLDV) homologues. It is found that the *P. patens* genes are clustered in DNA stretches (up to 13 kb) containing up to 16 NCLDV-like ORFs [218]. The acquisition of genomic segments by HGT has also been found in plants in relation to infections by pararetroviruses. With the use of HTS, two novel badnaviruses, which are dsDNA pararetroviruses of the family *Caulimoviridae,* were discovered in birch and chestnut and one unclassified *Caulimoviridae* member was discovered in pines. Pararetroviruses are often present as integrated, complete or fragmented and/or re-arranged genomic sequences in some host plant genomes and are then referred to as endogenous viral elements (EVEs). Some banana streak viruses (BSV) are found in episomal form with an endogenous counterpart (eBSV) [219]. In a few cases, badnavirus EVEs are known to be activated to generate viral infection (such as Banana streak OL, GF or IM viruses) [220]. Moreover, it is shown that genes of totiviruses and partitiviruses have widespread homologs in the nuclear genomes of eukaryotic organisms [221]. Evidence has been provided that some of the transferred genes are conserved and expressed in eukaryotic organisms, suggesting that these viral genes are also functional in the recipient genomes [221]. Whether the integration of the viral DNA of the novel caulimoviruses into the birch, chestnut and pine genome may play a role in its evolution remains to be untangled.

Viruses often coinfect single holobionts—in our study trees/shrubs—in nature. This was already known before the development of HTS technology, but the deep sequencing methods profoundly revealed the complexity of plants and tree viromes. Novel data on the birch virome clearly demonstrate in holobionts mixed infections by multiple plant viruses as well as multiple variants of the same virus species [14,15,189]. This is also the case in other non-forest plant species such as peach [222] or grapevine [223,224]. As a consequence, it is not easy to establish a correlation between such viral complexes and the appearance of symptoms or to differentiate symptomatology in cases of infection by a single virus or by two virus species. It is, however, suggested [189] that alterations in the viral communities in a holobiont may result in an alteration in symptom development.

Viruses often coinfect single fungal hosts in nature, and interesting virus–virus interplays in coinfected hosts have been reported that may be synergistic, apparently neutral or antagonistic [147]. Strong virus interference between unrelated RNA viruses was detected in *Cryphonectria parasitica*; for example, the (+)ssRNA virus Cryphonectria hypovirus 1 (CHV1) exerts a one-way synergistic effect on a co-infecting mycoreovirus 1 (MyRV1), resulting in enhanced virus accumulation and increased vertical transmission of MyRV1 [225]. In another interplay, the replication of Rosellinia necatrix victorivirus 1 (RnVV1) was strongly impaired by coinfection with MyRV1 or a mutant of CHV1 lacking the RNA silencing suppressor [170]. Recently, a unique mutualistic virus–virus interplay was reported, where the capsidless (+)ssRNA yado-kari virus (YkV1) was hosted by an unrelated dsRNA virus, yado-nushi virus (YnV1); while YnV1 could complete its replication cycle, YkV1 relied on YnV1 for its viability [226]. In another interaction system, the hypovirulence-associated mycoreovirus, named Sclerotinia sclerotiorum mycoreovirus 4 (SsMYRV4), could suppress host non-self-recognition and facilitate the horizontal transmission of heterologous viruses among vegetatively incompatible *S. sclerotiorum* individuals to create a bridge donor strain for mycovirus spread under natural conditions [227]. Such examples provide insight into the potential for broad-spectrum virus control mediated by RNA silencing.

Interspecific virus transmission is often suggested in mycoviruses [196,228]. In vitro experiments have shown that Cryphonectria hypovirus 1 was transmitted horizontally between the chestnut blight fungus *Cryphonectria parasitica*, and a sympatric unidentified *Cryphonectria* species via hyphal anastomosis [228]. Similarly, highly similar Heterobasidion RNA virus 1 (HetRV1) strains with 98% nucleotide level similarity were found from *H. parviporum* and *H. australe* growing in the same region in Bhutan, an observation that suggests recent virus transmission between these taxonomically distant *Heterobasidion* species in nature [131]. In another host, *Fraxinus* spp., hyphal anastomosis and transfer of the mitovirus Hymenoscyphus fraxineus mitovirus 1 (HfMV1) between both *Hymenoscyphus albidus* and *H. fraxineus* in ash is also hypothesized [196]. Bearing in mind that interspecies virus transmission is possible between viruses occurring in distantly related organisms, such as viruses infecting fungi, plants, oomycetes and invertebrates [229,230], this evolutionary potential should also be taken into consideration in view of the forest virome.

The efforts to untangle the complex world of the microbiota—virome interaction has been initiated for the human holobiont. Evidence is provided that bacteria aid in the antiviral response to certain viruses; however, occasionally, they may act as promoters of viral infection [231]. The present review constitutes a conceptual change by putting communities of microbiota and virome in a forest together. In this way, it becomes obvious how many possibilities for interactions are open, with all the consequences these may have for all “partners” within a holobiont. An important consequence could be that since the microbiome genome can adjust to environmental dynamics more rapidly and by more processes than the host genome, it can play a fundamental role in the adaptation and evolution of the holobiont [216].

The accumulation of knowledge regarding abundant mycoviruses in plant pathogenic fungi in forests offers new possibilities for pathogen control and management. It was previously found that endophytes could potentially be utilized as biocontrol agents in integrated pest and disease management [232]. Practical applications for forest protection based on endophytes are still rare. As stated above, the major mycoviral biocontrol agent used in field conditions is the Cryphonectria hypovirus 1 (CHV1), which significantly reduces the pathogenicity of the ascomycetous chestnut blight fungus (*Cryphonectria parasitica*) in Europe [167,168]. Moreover, in *C. parasitica*, the introduction of a partitivirus and a megabirnavirus originating from another host fungus, *Rosellinia necatrix*, was also shown to reduce host virulence [233,234]. Several *R. necatrix* viruses, including a fungal reovirus, also show potential for controlling their native host [235,236,237,238]. Additionally, Heterobasidion partitiviruses 13 and 15 have been shown to restrict host growth and alter the mycelial morphology of the conifer root rot fungi *H. annosum* and *H. parviporum* [169,200]. The recently generated data that are summarized in the present review could trigger further novel, modern, sustainable and environmentally friendly pest control applications.

In the present review, we did not address viruses of arthropods; however, it should be noted that baculoviruses of insect pests of temperate forest trees have been investigated for their biocontrol potential. The most prominent, which we mention here, are the nuclear polyhedrosis virus affecting *Lymantria dispar* on birches [239], oaks and poplars [240]; the Condylorrhiza vestigialis multiple nucleopolyhedrovirus affecting *Condylorrhiza vestigialis* on poplars [241]; the Orgyia leucostigma nucleopolyhedrovirus infesting *Orgyia leucostigma* on birches, firs and spruces [242,243]; and the Neodiprion abietis nucleopolyhedrovirus (NeseSNPV) affecting *Neodiprion abietis* on conifers [244]. As part of the effort to control the sawfly, NeseSNPV was introduced from Sweden into Canada and spread rapidly through the cohort and ultimately the population, resulting in the long-term suppression of *N. sertifer* [245]. To the best of our knowledge, HTS application has not contributed any further progress in this field.

Regarding vectors that transmit viruses, little is known. The only corresponding reference in the past was given in 1972 when Nienhaus detected tobamoviruses in Californian oak leaves [102] and experimentally assumed a virus transmissible fungus (*Sphaerotheca lanestris*). Vector studies of forest viruses were not reported until 2012 when Mielke and Mühlbach [16] highlighted the confirmed vector transmission by eriophyid mites (*Phytoptus pyri*) conclusively shown with several emaraviruses: European mountain ash ringspot-associated virus (EMARaV), fig mosaic virus (FMV), rose rosette virus (RRV), raspberry leaf blotch virus (RLBV) and pigeonpea sterility mosaic virus.

Healthy trees and healthy forests are translated into forests providing better regulating services (associated with natural catastrophes), maintenance/habitat services (increased biodiversity) and multiple ecosystem services (affecting humans and animals). However, the question arises as to how the “healthy forest virome” is defined, taking into consideration that virus–host relationships span the entire range from aggressive parasitism to mutualism. The corresponding question was previously posed concerning the “healthy human virome”. In humans, the same virus can be either a symbiont or a pathogen depending on conditions such as the health status and developmental stage of the host. Koonin et al. [246] suggest that the boundary between symbiotic and pathogenic viruses is fluid, such that members of the healthy virome can become pathogens under changing conditions. Rather, the mode of virus–host interaction is a function of multiple factors, including environmental conditions and the host–virus population structure. They conclude that the healthy virome is heterogeneous and consists of three distinct components: a. viruses that systematically enter the human organism but do not replicate in humans; b. viruses infecting prokaryotes and, possibly, unicellular eukaryotes that comprise the healthy human microbiome; and c. viruses that actually replicate and persist in human cells. Similarly to human viruses, the relationship between a particular virus and its plant host can be rarely, if ever, defined by a single regime. Reviewed data from this study show that in a single holobiont, viruses may coexist that a. enter a tree host through their endophytic host (Table 2) or interact with the tree via their ectomycorrhizal or saprotroph fungal host (Table 3); b. directly enter the host and act as pathogenic or latent viral agents (Table 1); and/or c. can potentially be integrated into their tree host as EVEs (badnaviruses; see discussion above). We are, however, at a stage where we gather knowledge about viral occurrence in forests but are still far from defining the criteria for a “healthy forest virome”. In light of approaching this definition, knowledge on different types of symbiotic viruses must catch up to that of pathogenic viruses.

The characterization of many novel—pathogenic or non-pathogenic—viruses in a short time raises the question of how to profit from these new data in order to avoid forest disease outbreaks in the future. It is possible for a pathogen to initiate an emerging infectious disease (EID)? According to the pathogen–host–environment interplay theory [247], emergence starts with an existing disease complex or pathogen–host–environment complex—based on paradigms from human and animal disease. The drivers of a pathogen’s emergence cause a shift in the overall pattern of the pathogen–host–environment interactions, leading to an EID event. Examples of drivers in regard to a forest comprise deforestation and logging, human encroachment of forests and game reserves and increased interspecies contacts at the wildlife/agriculture interface. Impending climate change may support the spread of forest pathogens and diseases and play a role in the dispersal of forest epidemics. Based on significant changes in the environment, alterations in the interactions within the holobiont may underlie future outbreaks of diseases. In light of the current detrimental and on-going COVID-19 epidemic in humans, we propose that a driver analysis in forest pathogens should be conducted similarly across the fields of human, animal and plant health.

## Figures and Tables

**Table 1:** **Plant viruses infecting tree/shrub hosts and cryptoviruses.** For each virus, the virus name, genus and family name, virus tree/shrub host(s), countries in which the virus was reported and the related references are given. Numbers in the first column represent ordinal numbers of the listed viruses. Viruses that were discovered by means of HTS methods are indicated by the note “HTS” next to their ordinal number.

	Virus Name	Genus, Family Name	Host Name (s)	Distribution ^+^	References
1	apple chlorotic leaf spot virus (ACLSV)	*Trichovirus, Beta*- *flexiviridae* (+)ssRNA	*Aesculus hippocastanum*, *S. aucuparia*	UK, Germany	[6,29]
2	apple mosaic virus (ApMV)	*Ilarvirus, Bromoviridae* (+)ssRNA	*Carpinus, Sorbus, Aesculus, Betula*	Canada, Europe	[30,31](*Sorbus, Aesculus, Betula, Carpinus*); [32] (*Betula*); [33] (*Rubus*); [34]
3	arabis mosaic virus (ArMV)	*Nepovirus, Seco*- *viridae* (+)ssRNA	*Acer, Fraxinus, Populus, Rubus*, *Betula*	Europe, USA	[35] (*Betula*), [36] (*Populus*); [37,38] (*Fraxinus*); [39] (*Rubus)*; [40,41] (*Acer*)
4 HTS	aspen mosaic-associated virus (AsMaV)	*Emaravirus, Fimo*- *viridae* (−)ssRNA	*Populus tremula*	Germany	[42]
5 HTS	birch leafroll-associated virus (BLRaV)	*Badnavirus*, *Cauli-* *moviridae* RT virus	*Betula*	Germany, Finland	[14,15]
6 HTS	birch idaeovirus (BIV)	*Idaeovirus, Mayoviridae* (+)ssRNA	*Betula*	Germany	[14]
7 HTS	birch capillovirus (BCV)	*Capillovirus, Betaflexiviridae* (+)ssRNA	*Betula*	Germany	[14]
8 HTS	birch carlavirus (BiCV)	*Carlavirus, Betaflexiviridae* (+)ssRNA	*Betula*	Germany	[14]
9	blueberry scorch virus (BlScV)	*Carlavirus, Betafle*- *xiviridae* (+)ssRNA	*Sambucus nigra*	Poland	[43,44]
10	brome mosaic virus (BMV)	*Bromovirus, Bromoviridae* (+)ssRNA	*Salix*		[45]
11	cherry leaf roll virus (CLRV)	*Nepovirus, Secoviridae* (+)ssRNA	*Betula, Aesculus, Fagus, Fraxinus, Sambucus, Sorbus, Ulmus armeniaca*	Europe, USA	[6] (*Fagus*); [46] (*Betula*); [29] (*Aesculus*); [47] (*Fraxinus*); [13,48] (*Betula*); [49,50] (*Sambucus*); [51] (*Sambucus nigra, Sorbus*); [52] (*Ulmus armeniaca, Fagus*); [53]
12	cherry rasp leaf virus (ChRLV)	*Cheravirus, Secoviridae* (+)ssRNA	*Sambucus nigra subsp. caerulea,*	USA	[54]
13 HTS	chestnut mosaic virus (ChMV)	*Badnavirus, Cauli-* *moviridae* RT virus	*Castanea sativa*	Italy, France	[55]
14 HTS	common oak ringspot-associated virus (CORaV)	*Emaravirus, Fimoviridae* (−)ssRNA	*Quercus robur*	Germany	[56,57]
15 HTS	elderberry aureusvirus 1 (ElAV1)	*Aureusvirus, Tombusviridae* (+)ssRNA	*Sambucus nigra*	Czech Republic	[58]
16-20 HTS	elderberry carlaviruses A,B,C,D,E (ElVA-ElVE)	*Carlavirus, Betaflexiviridae* (+)ssRNA	*Sambucus nigra*	USA	[59,60]
21 HTS	elm carlavirus (ECV)	*Carlavirus, Betaflexiviridae* (+)ssRNA	*Ulmus laevis*	Germany	[61,62,63]
22	elderberry latent virus (ELV)	*Pelarspovirus, Tombusviridae* (+)ssRNA	*Sambucus nigra, S. canadensis*	Austria, Poland, USA	[49,59]
23	Elm mottle virus (EMoV)	*Ilarvirus, Bromoviridae* (+)ssRNA	*Ulmus*	central Europe, Russia, UK	[64,65,66]
24 HTS	European mountain ash ringspot-associated virus (EMARaV)	*Emaravirus, Fimoviridae* (−)ssRNA	*Sorbus aucuparia, Aronia melanocarpa*, *Amelanchier, Karpatiosorbus × hybrid, S. intermedia*	Germany, Finland, Czech Republic, UK, Russia, Sweden, Poland, Norway	[67]; [18]; [68]; [17,69]; [70]; [19,71]; [72]; [73];
25 HTS	maple mottle-associated virus	*Emaravirus, Fimoviridae* (−)ssRNA	*Acer pseudoplatanus*	Germany	[74]
26	peanut stunt virus (PSV)	*Cucumovirus, Bromoviridae* (+)ssRNA	*Robinia pseudoacacia*	Croatia, Italy, Iran	[75,76,77,78]
27 HTS	Pinus nigra virus 1 (PnV1)	unclass. *Caulimoviridae* RT virus	air samples, *Pinus nigra*	Spain	[79]
28 HTS	Pinus patula amalgavirus 1	unclass. *Amalgaviridae* dsRNA	*Pinus patula*	TSA database	[80]
29	Pinus sylvestris partitivirus NL-2005	put. *Cryptovirus*, unclass. *Partitiviridae* dsRNA	*Pinus sylvestris*	Germany	[81]
30	poplar mosaic virus (PopMV)	*Carlavirus, Betafle*- *xiviridae* (+)ssRNA	*Populus*	UK, Germany	[82,83,84]
31 HTS	Sambucus virus S (SVS)	*Bromovirus, Bromo*- *viridae* (+)ssRNA	*Sambucus nigra*	Czech Republic	[85]
32	strawberry latent ringspot virus (SLRV)	*Stralarivirus*,*Secoviridae* (+)ssRNA	*Aesculus hippocastanum*, *Robinia pseu- doacacia*	Germany, Poland	[86,87,88,89]
33	tomato black ring virus (ToBRV)	*Nepovirus, Secoviridae* (+)ssRNA	*Sambucus nigra*, *Populus*	UK, Poland	[36,90]
34	tomato bushy stunt virus (TBSV)	*Tombusvirus, Tombusviridae* *(+)ssRNA*	*Sambucus nigra*, *S. canadensis*	USA, Czech Republic	[91,92]
35	tomato mosaic virus (ToMV)	*Tobamovirus, Virgaviridae* (+)ssRNA	*Picea rubens* *, P. mariana, Abies balsamea, Salix*	Canada, USA	[45,93,94]
36	tomato ringspot virus (ToRSV)	*Nepovirus*, *Secoviridae* (+)ssRNA	*Betula, Fraxinus*, *Populus*, *Ulmus americana*	UK, USA	[35,36,95,96]
37	tobacco mocaic virus (TMV)	*Tobamovirus, Virgaviridae* (+)ssRNA	*Fraxinus, Quercus*	USA, Hungary, Germany	[97,98,99,100,101,102,103,104]
38	tobacco necrosis virus (TNV)	*Alphanecrovirus, Tombusviridae*, (+)ssRNA	*Betula, Fagus, Fraxinus,Pinus*, *Quercus, Sambucus nigra*	Europe	[5,35,91,105,106]
39	tobacco rattle virus (TRV)	*Tobravirus, Virgaviridae* (+)ssRNA	*Fraxinus*, *Populus*	Germany	[107,108]
40	tobacco ringspot virus (TRSV)	*Nepovirus Secoviridae* (+)ssRNA	*Fraxinus*, *Sambucus nigra*	USA	[38,98,99,108,109,110,111,112]
41	white ash mosaic virus (WAMV)	unclassified *Flexiviridae* (+)ssRNA	*Fraxinus americana*	USA	[113,114]
42 HTS	putative cryptovirus	Partitiviridae dsRNA	*Fraxinus americana*		[115]
43 HTS	putative caulimovirus	Caulimoviridae RT virus	*Fraxinus americana*		[115]

^+^ The virus distribution data were retrieved from the Invasive Species Compendium CABI in combination with information from the publications referred to in the present review.

**Table 2:** Mycoviruses occurring in plant pathogenic fungi. For each virus, the virus name, genus and family name, fungal host(s), tree species, countries in which the virus was reported and the related references are given. Numbers in the first column represent ordinal numbers of the listed viruses. Viruses that were discovered by means of HTS methods are indicated by the note “HTS” next to their ordinal number.

	Virus Name	Genus, Family Name	Fungal Host (s)	Tree Host	Distribution ^+^	References
1 HTS	Armillaria borealis mycovirgavirus 1 (AbMV1)	unclass. *Virgaviridae* (+)ssRNA	*Armillaria borealis*	*Populus* spp.	Russia	[116]
2–4 HTS	Armillaria borealis ambi-like virus 1, 2, 3 (AbAlV1–3)	unclass. *Riboviria*	*Armillaria borealis*	*Populus* spp.	Russia, Finland	[116]
5 HTS	Armillaria mellea negative strand RNA virus 1 (AmNSRV1)	*Mymonaviridae* (−)ssRNA	*Armillaria mellea*	*Quercus robur*	South Africa	[116]
6 HTS	Armillaria mellea ourmia-like virus 2 (AmOlV2)	*Botourmiaviridae* (+)ssRNA	*Armillaria mellea*	*Quercus robur*	South Africa	[116]
7–11 HTS	Cronartium ribicola mitovirus 15 (CrMV1–5)	unclass. *Mitovirus, Mitoviridae* (+)ssRNA	*Cronartium ribicola*	*Pinus strobus*	North America	[117]
12–14	Cryphonectria hypovirus 1, 2, 3 (CHV-1–3)	*Hypovirus, Hypoviridae* (+)ssRNA	*Cryphonectria parasitica*	*Castanea* spp., *Aesculus hippocastanum*	England, Croatia, Slovenia, Greece, Turkey Slovakia, USA	[118,119,120,121,122,123,124]
15 HTS	Cryphonectria parasitica ambivirus 1- NB631 (CpaV1)	*Riboviria*; *unclass. Ambivirus* (−)ssRNA	*Cryphonectria parasitica*	*Castanea sativa*	Azerbaijan	[125]
16	Cryphonectria parasitica mitovirus 1 (CMV-1-cpNB631)	*Mitovirus,Mitoviridae* (+)ssRNA	*Cryphonectria parasitica*	*Castanea sativa*	USA	[126]
17 HTS	Cryphonectria parasitica sclerotimonavirus 1 (CpSV1)	unclass.*Sclerotimonavirus, Mymonaviridae* (−)ssRNA	*Cryphonectria parasitica*	*Castanea sativa*	Azerbaijan	[125]
18–20 HTS	Heterobasidion mitovirus 1, 2, 3 (HetMV1–3)	*Mitovirus*, *Mitoviridae* (+)ssRNA	*H. annosum and H. parviporum*	*Pinus sylvestris, Picea abies*	Poland, Finland, Russia	[9,127] [128]
21	Heterobasidion RNA virus 6 (HetRV6 *)	*Orthocurvulavirus, Curvulaviridae* dsRNA	*Heterobasidion abietinum, H. annosum, H. parviporum, H. occidentale*	*Abies alba, A. sibirica, A. cephalonica, A. cilicica, A. equi-trojani, A. concolor, Pinus sylvestris, P. nigra, P. obovata, Picea abies*, *Fagus*	Europe, USA	[129]
22–35	Heterobasidion partitivirus 1, 3, 4 *, 5, 9, 10, 11, 12 13 *, 14, 15, 16, 20 (HetPV1, HetPV3–5, HetPV9–16, HetPV20)	*Alphapartitivirus*, *Partitiviridae* dsRNA	*Heterobasidion abietinum*, *H. ecrustosum*, *H. parviporum*, *H. occidentale*, *H. australe*, *H. annosum*, *H. irregulare*	*Abies cephalonica*, *A. concolor*, *Pinus massoniana*, *P. wallichiana*, *P. sylvestris*, *P. elliottii*, *P. abies, P. pinea, Picea likiangensis*	Greece, China, Finland, Italy, Poland, Russia, USA, Bhutan	[127,130,131,132,133,134,135,136]
36–38	Heterobasidion partitivirus 2 *, 7 *, 8 (HetPV2–8)	*Betapartitivirus, Partitiviridae* dsRNA	*Heterobasidion parviporum, H. annosum, H. irregulare*	*Picea abies, P. sylvestris, P. pinea*	Finland, Italy	[127,134,137]
39	Hymenoscyphus fraxineus mitovirus 1 (HfMV1)	unclass. *Mitovirus, Mitoviridae* (+)ssRNA	*Hymenoscyphus fraxineus*	*Fraxinus* spp.	Switzerland, Japan, Poland, Germany, Lithuania, Norway	[138]
40–42 HTS	Fusarium circinatum mitovirus 1, 2–1 and 2–2 (FcMV1, FcMV2–1, FcMV2–2)	unclass. *Mitovirus, Mitoviridae* (+)ssRNA	*Fusarium circinatum*	*Pinus radiate, P. pinaster, P. nigra, P. sylvestris*	Spain	[139,140,141]
43–44	Gremmeniella abietina mitochondrial RNA virus S1, S2 (GaMRV-S1, S2)	*Mtovirus*, *Mitoviridae* (+)ssRNA	*Gremmeniella abietina*	mainly *Pinus sylvestris, Picea, Abies* and *Larix*	Northern and Central Europe, North America and Japan, Spain, Turkey	[142,143]
45	Gremmeniella abietina RNA virus L1 (GaRV-L1)	*Victorivirus*, *Totiviridae* dsRNA	*Gremmeniella abietina*	mainly *Pinus sylvestris, Abies* and *Larix*	Finland	[143]
46	Gremmeniella abietina RNA virus MS1 (GaRV-MS1)	*Gammapartitivirus*, *Partitiviridae* dsRNA	*Gremmeniella abietina*	*Pinus sylvestris, (Abies and Larix)*	Finland	[143]
47	Gremmeniella abietina RNA virus 6 (GaRV6)	*Curvulaviridae* dsRNA	*Gremmeniella abietina*	*Pinus halepensis*	Spain	[144]
48	Gremmeniella betaendornavirus (XL) (GBRV-XL)	*Endornaviridae* (+)ssRNA	*Gremmeniella abietina*	*Pinus sylvesris, P. contorta*	Finland	[145,146]
49	Gremmeniella fusarivirus 1 (GFV1)	unclass. Riboviria, proposed family “*Fusariviridae*” (+)ssRNA	*Gremmeniella abietina*	*Pinus hapepensis*	Spain	[146]
50–52	Mycoreovirus 1, 2, 3 (MyRV-1–3)	*Mycoreovirus*, *Reoviridae* dsRNA	*Cryphonectria parasitica*	*Castanea sativa, Prunus*		[147,148,149]
53–60	Ophiostoma mitoviruses 1a, 1b, 1c, 3a, 3b, 4, 5, 6 (OMV1a–OMV6)	*Mitovirus,* *Mitoviridae* (+)ssRNA	*Ophiostoma novo-ulmi*	*Ulmus*	UK, Canada	[150,151]
61 HTS	Phytophthora alphaendornavirus 1 (PEV1)	*Alphaendornavirus, Endornaviridae* (+)ssRNA	*Phytophthora ramorum, Phytophthora* taxon douglasfir	*Pseudotsuga menziesii, Quercus agrifolia*, *Viburnum* spp., *Laurus nobilis*, *Rhododendron*	USA, UK, Netherlands	[152]
62 HTS	Phytophthora cactorum RNA virus 1 (PcRV1)	unclass. *Totiviridae* dsRNA	*Phytophthora cactorum*	*Betula pendula*	Denmark	[153]
63 HTS	Sphaeropsis sapinea RNA virus 1 (SsRV1)	*Victorivirus, Totiviridae* dsRNA	*Diplodia pinea, D. scrobiculata*	*Pinus roxburghii*	South Africa	[154]

^+^ The virus distribution data were retrieved from the Invasive Species Compendium CABI in combination with information from the publications referred to in the present review. * Full-genome data for these viruses were generated by HTS (RNA-Seq or small RNA sequencing sRNA-Seq)) following their first discovery by traditional methods.

**Table 3:** Mycoviruses occurring in mutualistic fungi and saprotrophs. For each virus, the virus name, genus and family name, fungal host(s), tree species, countries in which the virus was reported and the related references are given. Numbers in the first column represent ordinal numbers of the listed viruses. Viruses that were discovered by means of HTS methods are indicated by the note “HTS” next to their ordinal number.

	Virus Name	Genus, Family Name	Fungal Host (s)	Tree Species	Distribution ^+^	References
1 HTS	Hygrophorus penarioides partitivirus 1 (HpPV1)	unclass. *Alpha*- *partitivirus, Partitiviridae* dsRNA	*Hygrophorus penarioides*	*Quercus petraea*	Turkey	[155]
2 HTS	Geopora sumneriana mitovirus 1 (GsMV1)	unclass. *Mitovirus, Mitoviridae*/*Narnaviridae* (+)ssRNA	*Geopora sumneriana*	*Cedrus libani*	Turkey	[156]
3 HTS	Gyromitra esculenta endornavirus 1 (GeEV1)	unclass. *Endornaviridae* (+)ssRNA	*Gyromitra esculenta*	*Pinus brutia*	Turkey	[157]
4 HTS	Gyromitra esculenta partitivirus 1 (GePV1)	unclass. Partitiviridae dsRNA	*Gyromitra esculenta*	*Pinus brutia*	Turkey	[158]
5	Lactarius rufus RNA virus 1 (LrRV1)	*Orthocurvulavirus, Curvulaviridae* dsRNA	*Lactarius rufus, L. tabidus*	*Pinus sylvesrtris, Picea abies*	Finland	[159]
6	Lactarius tabidus RNA virus 1 (LtRV1)	*Orthocurvulavirus, Curvulaviridae* dsRNA	*Lactarius rufus, L. tabidus*	*Pinus sylvesrtris, Picea abies*	Finland	[159]
7 HTS	Picoa juniperi mycovirus 1 (PjMTV1)	unclass. *Riboviria*, newly proposed Megatotiviridae dsRNA	*Picoa juniperi*	various forest tree species	Turkey	[160]
8	Tuber aestivum virus 1 (TaV1)	*Totivirus, Totiviridae* dsRNA	*Tuber aestivum*	mixed beech forest	Hungary	[161]
9	Tuber aestivum endornavirus (TaEV)	*Betaendornavirus, Endornaviridae* (+)ssRNA	*Tuber aestivum*	mixed oak forest	Hungary	[162]
10	Tuber aestivum mitovirus (TaMV)	unclass. *Mitovirus*, *Mitoviridae* (+)ssRNA	*Tuber aestivum*	mixed oak forest	Hungary	[163]
11	Tuber excavatum mitovirus (TeMV)	unclass. *Mitovirus, Mitoviridae* ssRNA(+)	*Tuber excavatum*	mixed beech forest	Germany	[164]
12–13 HTS	putative alpha- and betapartitiviruses (ScPV)	*Partitiviridae* dsRNA	*Sarcosphaera coronaria*	*Pinus brutia*	Turkey	[165]

^+^ The virus distribution data were retrieved from the Invasive Species Compendium CABI in combination with information from the publications referred to in the present review.

## Abbreviations

aa	amino acid
BLRD	birch leaf-roll disease
DOP-PCR	degenerate oligonucleotide-primed PCR
dsRNA	double-stranded RNA
ECM	ectomycorrhizal fungi
EID	emerging infectious disease
EVE	endogenous viral element
GPP	glycoprotein precursor
HTS	high-throughput sequencing
ICTV	International committee on taxonomy of viruses
ISID	International Society for Infectious Diseases
IUCN	International Union for Conservation of Nature
(−)RNA	negative sense RNA
N	nucleocapsid protein
NCLDV	nucleocytoplasmic large DNA viruses
ORF	open reading frame
(+)RNA	positive sense RNA
ProMED	Program for Monitoring Emerging Diseases
RdRP	RNA-dependent RNA polymerase
RCRE	rolling-circle replication endonucleases
RT	reverse transcriptase
RT virus	reverse-transcribing virus
ssDNA	single-stranded DNA
SSC	Species survival commission
TSA	Transcriptome Shotgun Assembly
unclassified	unclass.
